# Small extracellular vesicles proteome reveals persistent inflammatory and coagulopathic dysregulation in long-COVID

**DOI:** 10.3389/fimmu.2026.1805159

**Published:** 2026-07-20

**Authors:** Sivasankar Chandran, Ling Chen, Anil Kumar Ram, Leslie Spikes, Prabhakar Chalise, Navneet K. Dhillon

**Affiliations:** 1 Division of Pulmonary and Critical Care Medicine, Department of Internal Medicine, Kansas City, KS, United States; 2 Department of Biostatistics & Data Science, University of Kansas Medical Center, Kansas City, KS, United States

**Keywords:** acute-COVID-19, extracellualr vesicles, PASC, plasma, proteomics

## Abstract

**Background:**

Post-acute sequelae of SARS-CoV-2 (PASC) or Long-COVID affects millions and remains mechanistically undefined due to its heterogeneous clinical presentation. Identifying robust biological signatures is essential for understanding disease mechanisms and improving diagnosis. Here, we investigated the protein cargo of plasma-derived small extracellular vesicles (SEVs) from PASC-positive and PASC-negative individuals to identify EV-linked biomarkers of Long-COVID.

**Methods:**

SEVs were isolated from EDTA plasma of PASC-positive (n=20) and PASC-negative (n=11) individuals using size-exclusion chromatography. SEV protein cargo was profiled across more than 5400 proteins using the Olink Explore HT platform.

**Results:**

PASC-positive patients commonly reported fatigue, shortness of breath, brain fog, sleep disruption, and mood changes. Proteomic analysis revealed 269 significantly dysregulated proteins, including 84 upregulated and 21 downregulated, with a fold change >2 in PASC. These differentially altered proteins were enriched in pathways related to coagulation, inflammation, apoptosis, fibrosis, extracellular matrix remodeling, mitochondrial dynamics, and immune activation. PASC-positive SEVs showed persistent increases in FN1, HCF-H, HGF, and IL-17RA, proteins previously dysregulated in acute COVID-19. These markers showed greater differences in SEVs than in matched plasma, particularly HGF and IL-17RA, which were significantly altered in SEVs but not in plasma.

**Conclusion:**

Proteomic alterations in SEVs from PASC patients highlight the inflammatory, thrombotic, and neurobiological dysregulation, underscoring the potential of SEVs as biomarkers and mechanistic drivers of long COVID.

## Introduction

Regardless of the severity of the severe acute respiratory syndrome coronavirus-2 (SARS-CoV-2) infection, studies indicate that approximately 45% of COVID-19 survivors continue to exhibit post-acute sequelae of SARS-CoV-2 infection (PASC), commonly referred to as long COVID (1). More recently, a 2025 meta-analysis estimated that the global pooled prevalence of long COVID is about 36% among individuals with confirmed COVID-19 (2). The short- and long-term consequences of PASC markedly affect patients’ quality of life, productivity, and overall health care expenditures (3). The clinical presentation of PASC varies widely and therefore represents a significant challenge for both clinical management and public health (4) (5).

PASC is characterized by a broad spectrum of symptoms affecting multiple physiological systems, with over 100 distinct manifestations reported. As a multiorgan condition, PASC frequently presents with persistent pulmonary symptoms such as dyspnea, reduced exercise tolerance, hypoxemia, and restrictive ventilatory defects (6). Neuropsychiatric manifestations such as fatigue, cognitive deficits (brain fog), anxiety, and sleep disturbances are also frequently observed (7, 8). Cardiovascular manifestations, including palpitations, chest discomfort, and myocardial impairments, further contribute to the clinical burden (9, 10). In addition, endocrine (11, 12) renal, gastrointestinal, hepatobiliary, and dermatologic abnormalities, highlight the multisystemic nature of post-acute sequelae of COVID-19 (13). Several interrelated pathogenic mechanisms have been proposed to underlie the development of long COVID, with the most prominent including immune dysregulation, viral persistence, or the continued presence of viral components, autoimmune responses, and endotheliopathy (5).

Extracellular vesicles (EVs) released by cells in response to multiple physiological and pathological stimuli, such as infection, stress, and inflammation, play a crucial role in disease pathogenesis (14–18). EVs carry bioactive molecules such as proteins, lipids, and microRNAs that have the ability to modulate inflammation, coagulation, vascular remodeling, and immune responses (19, 20). EV cargo is increasingly recognized as a valuable source of biomarkers for monitoring disease progression with improved detection of subtle molecular alterations. Compared with whole−blood analytes, plasma−derived EV contents may serve as better biomarkers because they are less complex, shielded from degradation, and remain stable for extended periods (21, 22).

EVs have already gained considerable importance as informative biomarkers in acute COVID-19, reflecting both viral persistence and host immune responses (23). Emerging evidence indicates that EV profiles differ between mild and severe disease and are closely associated with inflammation and immune dysregulation, supporting their utility in assessing disease severity (14). Our previous proteomic analysis of EVs from patients with acute COVID-19 showed enrichment of proinflammatory, procoagulant, immunoregulatory, and tissue remodeling proteins, clearly distinguishing symptomatic patients from uninfected controls with matched comorbidities (17). Furthermore, EV cargo differed between moderate and critical disease severity. Other studies have similarly highlighted EVs as a promising liquid biopsy platform for integrated diagnosis and monitoring of COVID-19 disease progression (14, 24). In this study, we now investigate the protein cargo in circulating small EVs (SEVs) from PASC positive patients to identify EV-associated biomarkers indicative of Long-COVID and further understand the pathophysiology of PASC.

## Materials and methods

### Human samples and data collection

Participants classified as having post−acute sequelae of SARS−CoV−2 infection (PASC) (N = 20) were those who reported one or more ongoing symptoms persisting beyond 6-9 months after their initial infection, except one participant infected 66 days prior, and two others infected 85 and 89 days before, respectively. Symptom persistence was identified either through responses to a REDCap survey or documentation of continued symptoms during clinical follow−up. Reported symptoms included fatigue, post−exertional malaise, dyspnea, cough, anosmia or ageusia, fever, myalgias, headaches, chest discomfort, cognitive difficulties (“brain fog”), sleep disturbances, mood changes, rash, and tinnitus. Individuals without PASC were selected from participants in the ACTIV−2 clinical trial who had completed the first 24 weeks of study follow−up. These participants had a confirmed prior SARS−CoV−2 infection (PCR−positive) 6-15 months before assessment and reported no persistent symptoms (N = 11). One PASC negative individual, however, underwent a blood draw at 84 days after acute infection. For validation analyses, plasma samples were also obtained from hospitalized acutely infected patients with a confirmed positivity for COVID-19 PCR (N = 10) and from healthy volunteers whose samples were collected before the COVID−19 pandemic (N = 5). All participants were enrolled through The University of Kansas Health System (TUKHS) COVID-19 Biorepository, and blood samples were collected in accordance with the protocols approved by the Institutional Review Board. Demographic characteristics, comorbidities, body mass index, interval from last positive test to study enrollment, and symptom profiles were obtained from electronic medical records or participant surveys and stored in a secure database. Survey data were collected using REDCap and incorporated the standardized World Health Organization (WHO) Global COVID−19 Clinical Platform Case Report Form for Post−COVID Conditions. Participants completed surveys at enrollment and at 3−month intervals thereafter.

### Isolation of small EVs from plasma samples

EDTA plasma was separated within 4 hours of collection by centrifugation at 2000 × *g* for 15 minutes at 4 °C, aliquoted immediately, and stored at −80 °C until analysis. Plasma turbidity was monitored; in cases of hemolysis, it was documented, and such samples were excluded from EV isolation. Approximately 500 µl of frozen EDTA plasma (without prior freeze–thaw cycles) was thawed at room temperature and centrifuged at 2500 × *g* for 15 min at room temperature to obtain platelet-free plasma (PFP). The PFP was subsequently centrifuged at 20,000 × *g* for 15 min at 4 °C to pellet large extracellular vesicles, which were removed and stored separately. The resulting supernatant was subjected to small EV (SEV) isolation via size-exclusion chromatography (qEV original 35 nm columns; Izon Science, Cambridge, MA) as previously reported (17). Final SEV preparation was reconstituted in the same volume as the starting plasma volume used for isolation of EVs. NanoSight nanoparticle tracking analysis (NTA) was used to identify EV-enriched fractions, and fractions 7–10 were pooled. Pooled SEV fractions were concentrated to a final volume of 500 µl using Amicon Ultra-4 centrifugal filters (10 kDa; Millipore Sigma, USA).

### Characterization of SEVs

To assess the size distribution and concentration of SEVs, NTA was performed in each sample as described previously (17). Samples were diluted 1:100 in phosphate-buffered saline (PBS) and gently vortexed before analysis. The diluted suspensions were then introduced into the sample chamber using a syringe pump. For each sample, five 60-second videos were recorded using NanoSight software (version NTA 3.4) with screen gain set to 4 and camera gain to 10 during acquisition. A detection threshold of 10 and screen gain of 10 were used for data processing. Manual focusing was performed to optimize visualization and maximize SEV detection.

Isolated SEVs were lysed using a RIPA lysis buffer for Western Blot analysis of EV markers. Approximately 5 μg of SEV lysates were resolved on a 12% SDS-PAGE gel and subsequently transferred onto an Immobilon-P PVDF membrane. The membranes were incubated overnight at 4 °C with primary antibodies against established EV markers, including the tetraspanins: CD9, CD81, CD63; Integrin β1; and cytosolic proteins: TSG101 and Alix. HRP-conjugated IgG secondary antibodies were used for detection. Signals were developed using the Pierce ECL and SuperSignal West Femto (Thermo Fisher Scientific, USA), and the images were captured with the LI-COR Odyssey Fc imaging system.

### Olink proximity extension analysis

An equal volume of SEV sample from each PASC-positive (n=13) and PASC-negative (n=10) individual was lysed for Olink analysis using a proteomic-grade lysis buffer as described in previous studies (17, 25). Fifty microliters of lysed SEVs was then transferred in a randomized order to a 96-well PCR plate and submitted to the Clinical Genomics Center at the Oklahoma Medical Research Foundation for analysis. SEV protein cargo was profiled using the Olink Explore HT panels. Samples were randomly distributed across the plate, and both assay performance and sample quality were monitored using four internal controls. Protein abundance was reported as normalized protein expression (NPX) values on a log_2_ scale, where higher NPX values correspond to higher protein expression levels. Samples failing technical quality metrics or exhibiting abnormally high variability were excluded from further analysis. For validation, ELISA kits were obtained from Proteintech (Catalog numbers KE00788; KE00168, KE00900; KE00039) and the experiments were performed as per the manufacturer’s instructions.

### Statistical analysis

Differences in protein expression NPX values between PASC-positive and PASC-negative groups were assessed using linear models for omics data implemented in the *limma* Bioconductor R package (26). The Wilcoxon Rank Sum test was used to compare protein levels between participants with or without binary clinical symptoms such as shortness of breath, brain fog, and sleep disturbance. The protein level changes were visualized using heatmaps, and the differential expression analyses were summarized with volcano plots. All protein expression analyses were performed using R software version 4.5.0. For the SEV concentration measurement, Student’s t-test analysis was carried out using GraphPad Prism 9. A p-value <0.05 was considered statistically significant.

### Pathway enrichment analysis

Gene Ontology (GO) and Kyoto Encyclopedia of Genes and Genomes (KEGG) pathway enrichment analyses were performed using the Enrichr platform (https://maayanlab.cloud/Enrichr/). Canonical pathway analysis was conducted using Ingenuity Pathway Analysis (IPA; Qiagen), and Reactome pathway mapping was carried out using STRING database version 12.0, followed by visualization and network confirmation using Cytoscape. All bioinformatics analyses were conducted using the respective tools and databases available as of August 2025.

## Results

### Demographic and clinical characteristics

Demographic and clinical characteristics of participants whose plasma-derived SEVs were analyzed by PEA proteomics are summarized in **Table 1**. Age, sex, vaccination status, and smoking status did not differ between the groups. Although BMI values were slightly higher in PASC-positive participants compared with PASC-negative controls, the difference was not statistically significant. Similarly, the interval between the initial SARS-CoV-2 positive test and study enrollment appeared shorter among PASC-positive individuals; however, this difference was also not statistically significant. Pulmonary disease, depressive disorder, and GERD were more common among PASC-positive participants, whereas coronary artery disease was observed only in PASC-negative individuals, as shown in **Table 1**. Other conditions, such as diabetes and hypertension, occurred at comparable frequencies across groups. Within the PASC-positive cohort, the most frequently reported persistent symptoms included fatigue (76.9%), shortness of breath (69.2%), brain fog (46.2%), sleep disturbances (38.5%), musculoskeletal or chest pain (46.2%), and rash (38.5%), consistent with established clinical features of post-acute sequelae of SARS-CoV-2 infection.

**Table 1:** Demographic and clinical characteristics of individuals with and without PASC.

Characteristics	PASC positive (N = 13)^1^	PASC negative (N = 10)^1^	P value
Age (years)	44, (32-56)	57.5, (45-67)	0.11
Male	6 (46%)	5 (50%)	
Female	7 (54%)	5 (50%)	
BMI	30.13 (26.7-38.1)	30 (26.1-32.7)	0.4
Days between positive test and study enrollment	180 (126-221)	261 (181-319)	0.8
Hospitalized during an acute infection	6 (46.15%)	4 (50.00%)	>0.9999
Days of Hospitalization (if applicable)	4-29	2-35	0.8
COVID-19 Vaccinated	9 (69.23%)	6 (60.00%)	0.685
Boosted	6 (46.15%)	4 (57.14%)	0.6802
*Co-morbidities*			
Diabetes	2 (15.38%)	2 (25.00%)	>0.9999
Hypertension	4 (30.77%)	2 (25.00%)	0.66
Congestive Heart Failure	1 (7.69%)	1 (12.50%)	>0.9999
Coronary Artery Disease	0 (0.00%)	3 (37.50%)	0.0678
Kidney Disease	1 (7.69%)	1 (12.50%)	>0.9999
Pulmonary Disease	5 (38.46%)	1 (12.50%)	0.179
Depressive Disorder	4 (30.77%)	1 (12.50%)	0.3394
Anxiety	3 (23.08%)	0 (0.00%)	0.2292
GERD	4 (30.77%)	1 (12.50%)	0.3394
Smoking Status	1 (7.7%)	0 (0.00%)	>0.9999
Hyperlipidemia, Hypercholesterolemia, or Dyslipidemia	3(23.08%)	1 (12.50%)	0.6036
Migraines	3 (23.08%)	0 (0.00%)	0.2292
*PASC symptoms*			
Fatigue	10 (76.92%)	NA	
Post-exertional malaise	3 (23.08%)	NA	
Shortness of Breath	9 (69.23%)	NA	
Coughing	2 (15.38%)	NA	
Loss of taste or smell	4 (30.77%)	NA	
Fever	1 (7.69%)	NA	
Body aches, headaches, chest pain, stomach pain	6 (46.15%)	NA	
Brain Fog	6 (46.15%)	NA	
Sleep Disturbances	5 (38.46%)	NA	
Mood Changes	2 (15.38%)	NA	
Rash	5 (38.46%)	NA	

^1^ n (%); Median (IQR).

### Plasma-derived SEV characterization reveals no differences in particle number or size between PASC-positive and PASC-negative individuals

**Figure 1:**
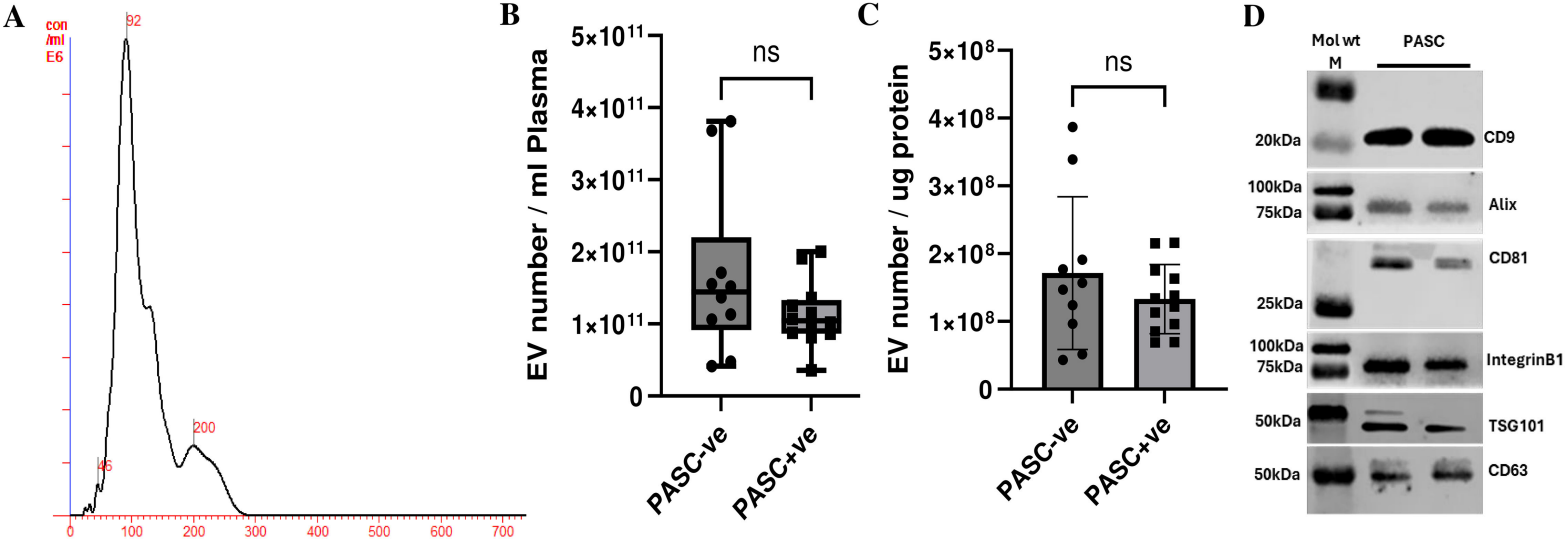
Characterization of plasma-derived small extracellular vesicles (SEVs) from PASC-positive (n = 13) and PASC-negative (n = 10) patients. SEVs were isolated from 0.5 ml of platelet-free EDTA plasma. **(A)** A representative line diagram of EV size distribution and **(B)** Nanoparticle tracking analysis (NTA) of total SEV counts expressed as particles per ml of the final EV suspension; and **(C)** SEV counts per ug of EV protein in each group using NanoSight nanoparticle tracking analyzer. **(D)** Representative western blot images showing EV-specific markers in the EV lysate from PASC samples.

SEVs isolated by size exclusion chromatography from PASC-positive and PASC-negative plasma samples were evaluated for particle concentration and size distribution. Particle size distributions were comparable between the groups, with the majority of SEVs falling within the 50-200 nm range, as illustrated in **Figure 1A**. Total SEV concentration, normalized per milliliter of plasma or per microgram of EV protein, also showed no significant differences between the groups (**Figures 1B, C**). Western blotting confirmed the presence of canonical EV markers, including tetraspanins, CD9, CD81, and CD63, as well as Integrin β1, Alix, and TSG101 (**Figure 1D**).

### SEV protein cargo exhibits significant alterations in PASC-positive individuals

**Figure 2:**
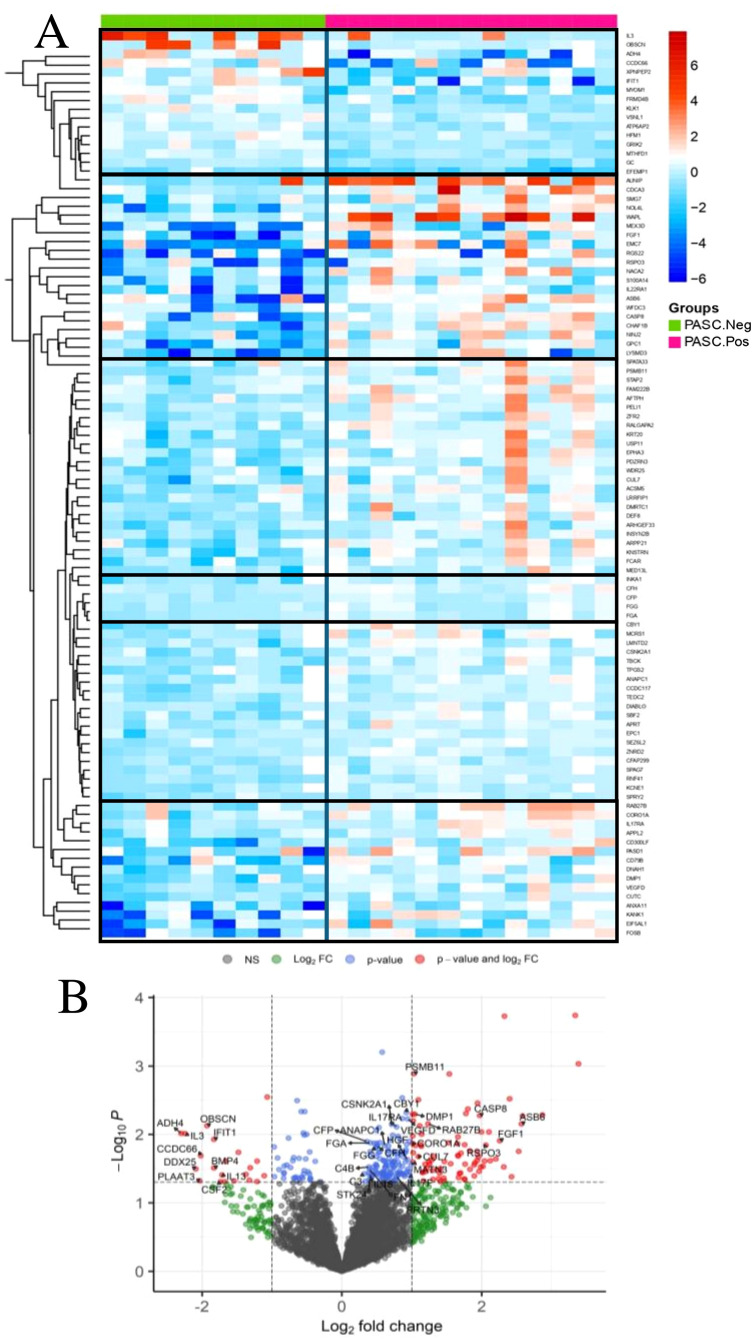
Proximity extension analysis (PEA) of circulating SEVs in PASC patients. EDTA plasma samples from PASC-positive (n = 13) and PASC-negative (n = 10) individuals were analyzed to compare SEV-associated protein cargo using Olink’s multiplex PEA technology. **(A)** A heatmap with hierarchical clustering displays significantly altered EV proteins between the two groups (P<0.05). **(B)** Volcano plots illustrate pairwise *post hoc* comparisons of SEV protein cargo, with −log10 (P value) plotted against the mean difference. Differentially altered proteins with *P* < 0.05 are highlighted in red. Positive and negative log 2 fold change values indicate upregulated and downregulated proteins, respectively.

Proteomic profiling using the Olink Explore HT platform identified substantial differences in SEV-associated proteins between PASC-positive (n = 13) and PASC-negative (n = 10) groups. Heatmaps illustrate differential protein expression patterns between groups (**Figure 2A**), with pronounced alterations in the proteins related to prothrombotic activity, apoptosis, cell proliferation, complement activation, and inflammatory signaling. The heatmap highlights differential SEV protein expression between groups. Overall, six distinct clusters of protein expression patterns were identified. Notably, one cluster on the top showed a consistent downregulation of proteins in the PASC-positive group, while the remaining five clusters exhibited predominant upregulation of SEV protein cargo in PASC-positive individuals.

A volcano plot summarizing the differential expression results revealed 269 significantly dysregulated proteins (P < 0.05) out of 5,416 analyzed, among which 210 proteins were at higher levels, while 59 were reduced in the PASC-positive group (**Figure 2B**). Notably, 17 proteins demonstrated >4 fold increase, including WAPL (WAPL cohesion release factor), AUNIP (aurora kinase A and ninein interacting protein), RGS22 (regulator of G protein signaling 22), MEX3D (mex-3 RNA binding family member D), ASB6 (ankyrin repeat and SOCS box containing 6), and EMC7 (ER membrane protein complex subunit 7). Thirteen proteins exhibited fold decrease below 0.58, including ADH4 (alcohol dehydrogenase 4, class II, pi polypeptide), IL3 (interleukin 3), DDX25 (DEAD-box helicase 25), PLAAT3 (phospholipase A and acyltransferase 3), and CCDC66 (coiled-coil domain containing 66). On the contrary, proteomic profiling of SEVs also revealed significantly reduced levels of CSF2 (GM-CSF), IL-3, and IL-13 in the PASC-positive group compared to the PASC-negative group. IFIT1 (Interferon-induced protein with tetratricopeptide repeats 1) was one of the most prominently downregulated proteins and is a major mediator of antiviral immune responses.

### Functional enrichment reveals dysregulated coagulation, complement, and inflammatory pathways in PASC

**Figure 3:**
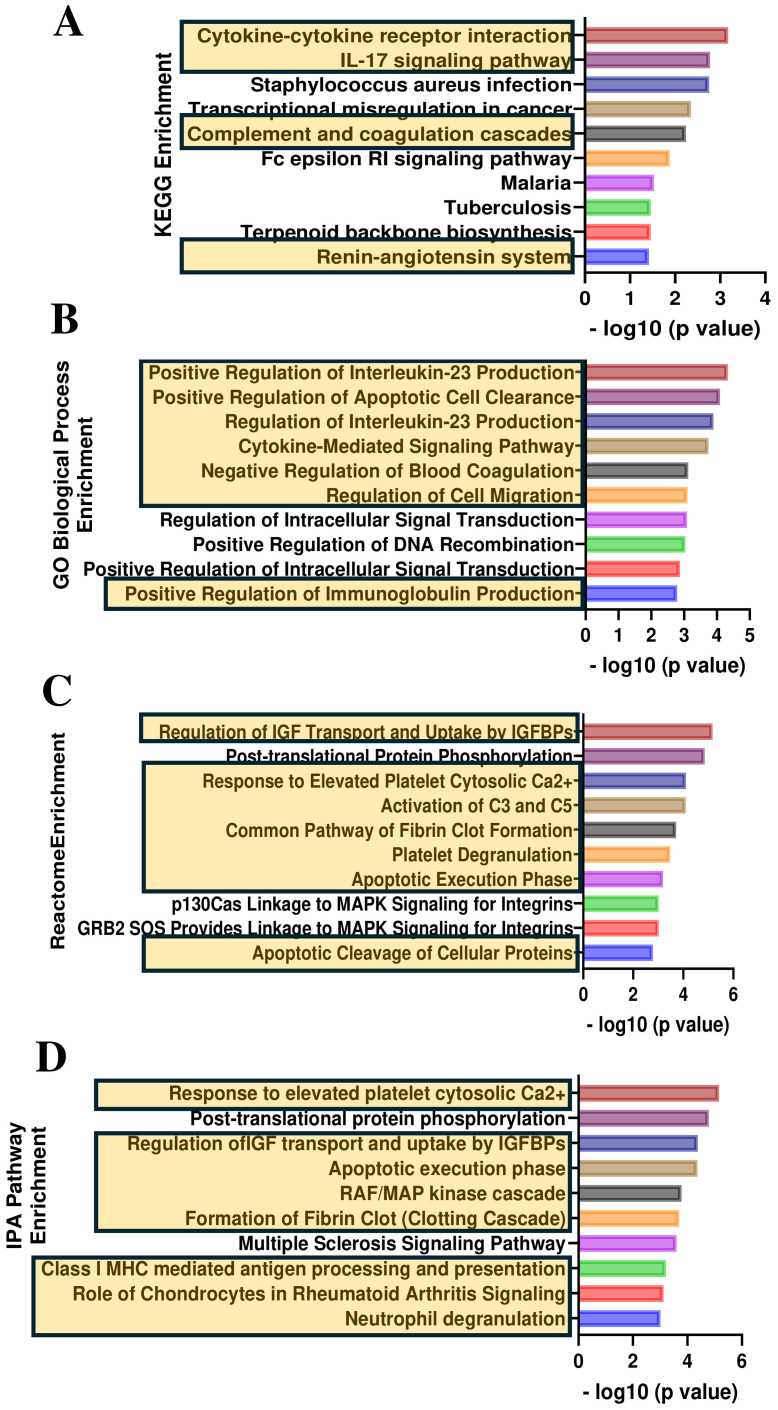
The top 10 significantly enriched pathways of significantly altered SEV-proteins between PASC-positive and PASC-negative groups (P<0.05) based on **(A)** KEGG, **(B)** Gene ontology biological process, **(C)** REACTOME, and **(D)** IPA analysis. Relevant biological processes are highlighted in yellow.

**Figure 4:**
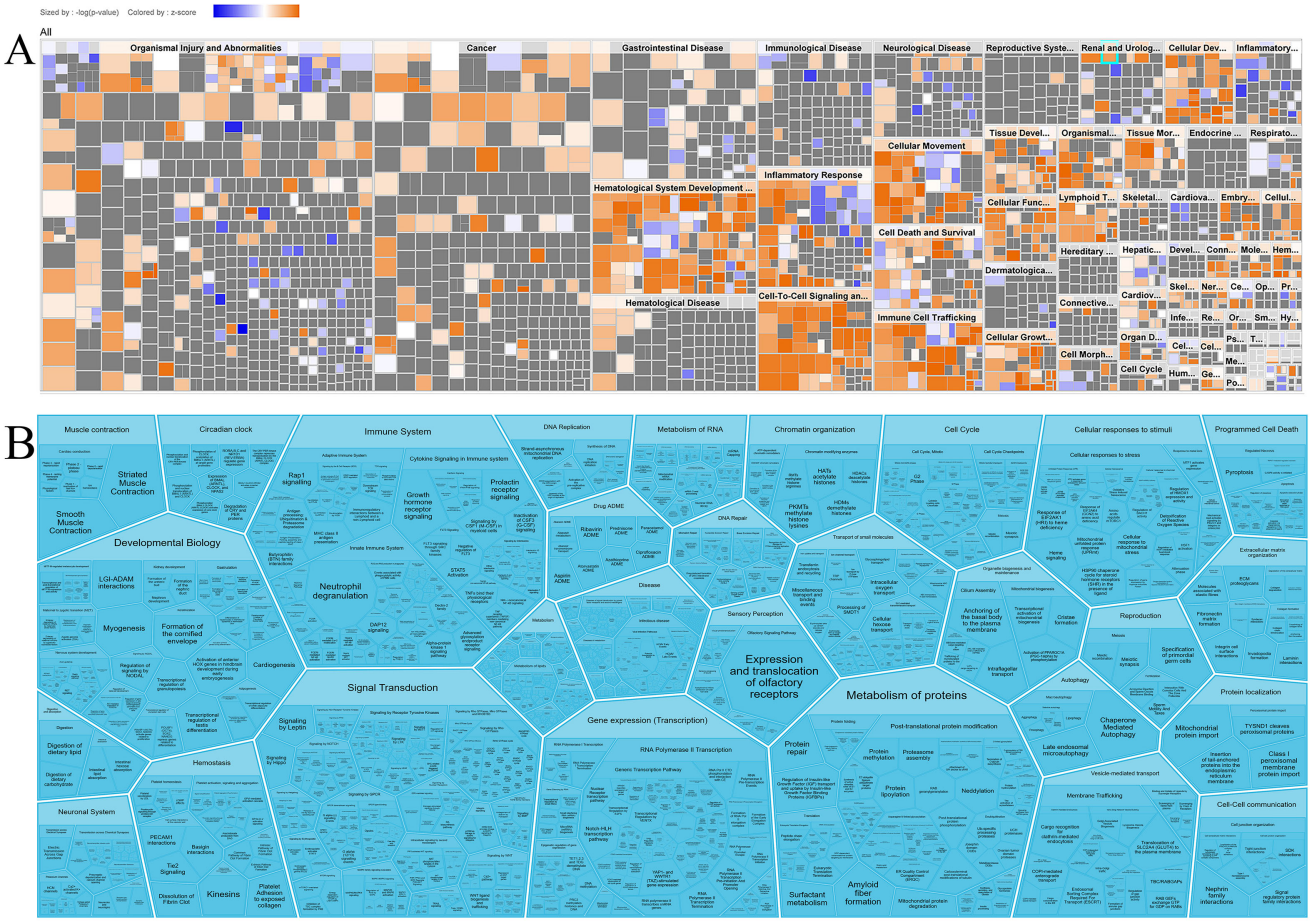
**(A)** IPA disease and function plot and **(B)** Voronoi diagram from the REACTOME tool depicting the interactions and relationships of the 269 significantly dysregulated proteins (P<0.05) between PASC-positive and PASC-negative groups. It shows predicted biological functions, diseases, and pathways. Each polygon/square (tile) represents a function or pathway, with the tile size proportional to the number of associated proteins. Tiles positioned closer together indicate functions that share molecular overlaps or are biologically related. **(A)** In IPA Colors reflect predicted activity states, where orange/red indicates activation, blue denotes inhibition, and white/gray corresponds to no significant prediction.

**Figure 5:**
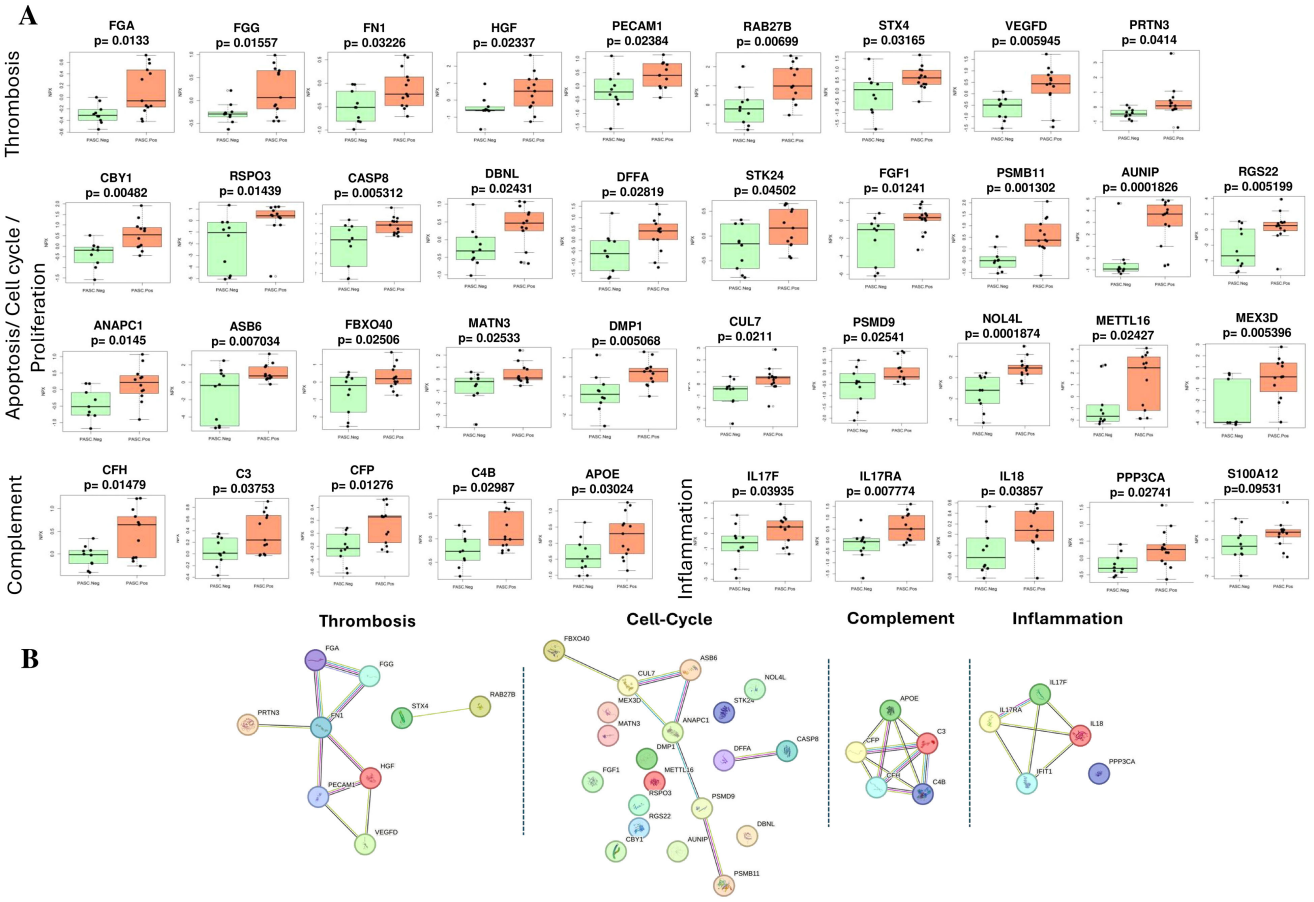
**(A)** Box-and-whisker plots showing differences in the levels of SEV-associated proteins related to inflammatory, thrombotic, apoptotic, and fibrotic pathways between PASC -positive and -negative groups. Plot displays normalized protein expression (NPX) on a log2 scale. The boxes represent the interquartile range (Q1–Q3) with the median shown as a line within the box, while the whiskers extend up to 1.5 times the IQR. Corresponding P values are indicated above each plot. **(B)** STRING protein-protein interaction network illustrating molecular interactions among the identified targets. Nodes denote proteins, while lines represent functional and physical associations derived from experimental evidence, curated databases, and predicted interactions.

Functional enrichment analyses using KEGG, GO, Reactome, and IPA databases consistently identified significant dysregulation of inflammatory, thrombotic, and complement pathways among altered SEV-associated proteins. Pathways related to apoptosis, immune regulation, and cell migration were also enriched, suggesting persistent immune activation and impaired tissue homeostasis(**Figures 3A–C**, **Figure 4B**). IPA further highlighted dysregulation of apoptosis/cell-cycle regulation, fibrin clot formation, and immune activation/inflammation signaling cascades. (**Figures 3D**, **4A**). **Figure 5A** shows a comparison of NPX values between groups of markedly elevated proteins in SEVs from PASC positive group implicated in coagulation and thrombosis, such as FGA, FGG, FN1, PECAM1, HGF, RAB27B, STX4, VEGFD, and PRTN3. Additional increased proteins associated with apoptosis, and Wnt signaling (CBY1, RSPO3, CASP8, DBNL, DFFA, STK24, and FGF1) and those involved in cell-cycle regulation or proliferation (ANAPC1, ASB6, FBXO40, MATN3, DMP1, CUL7, PSMD9, and PSMB11) are also shown across groups. Complement components, CFH, C3, CFP, and C4B, showing elevated levels in the PASC-positive group, highlight the complement activation as one of the major immunological features. Increased IL17F, IL17RA, IL18, and PPP3CA levels in the PASC group further indicate heightened pro-inflammatory cytokine signaling. S100A12 (ENRAGE), which we previously reported as one of the top-ranked inflammatory proteins associated with COVID-19 disease severity and prolonged hospitalization, also remained persistently elevated in SEVs from PASC-positive individuals. However, this increase did not reach statistical significance, likely due to the small sample size and the heterogeneity of symptoms within the PASC-positive cohort. STRING network analysis demonstrated extensive molecular interconnectivity among differentially expressed proteins (**Figure 5B**). Collectively, these results indicate that SEVs from PASC patients are enriched for proteins involved in immune and inflammatory signaling, coagulation cascades, apoptosis, and cell-cycle regulation.

### Symptom-associated SEV proteomic signatures in PASC-positive individuals

Exploratory Wilcoxon rank-sum analyses, without multiple testing adjustments, identified four proteins: NOL4L, STK24, PRTN3, and IL17RA, significantly associated with self-reported brain fog in PASC-positive individuals, suggesting potential involvement of inflammatory signaling pathways relevant to neurocognitive impairment. Sleep disturbance was associated with IFIT1, AUNIP, METTL16, PPP3CA, and RGS22 proteins implicated in immune activation, complement activation, cell cycle regulation, and coagulation. These associations highlight broad systemic immune and metabolic perturbations contributing to the symptom burden in PASC.

### Validation and comparison of PASC-associated SEV protein signatures with acute COVID-19 SEVs and matched plasma

**Figure 6:**
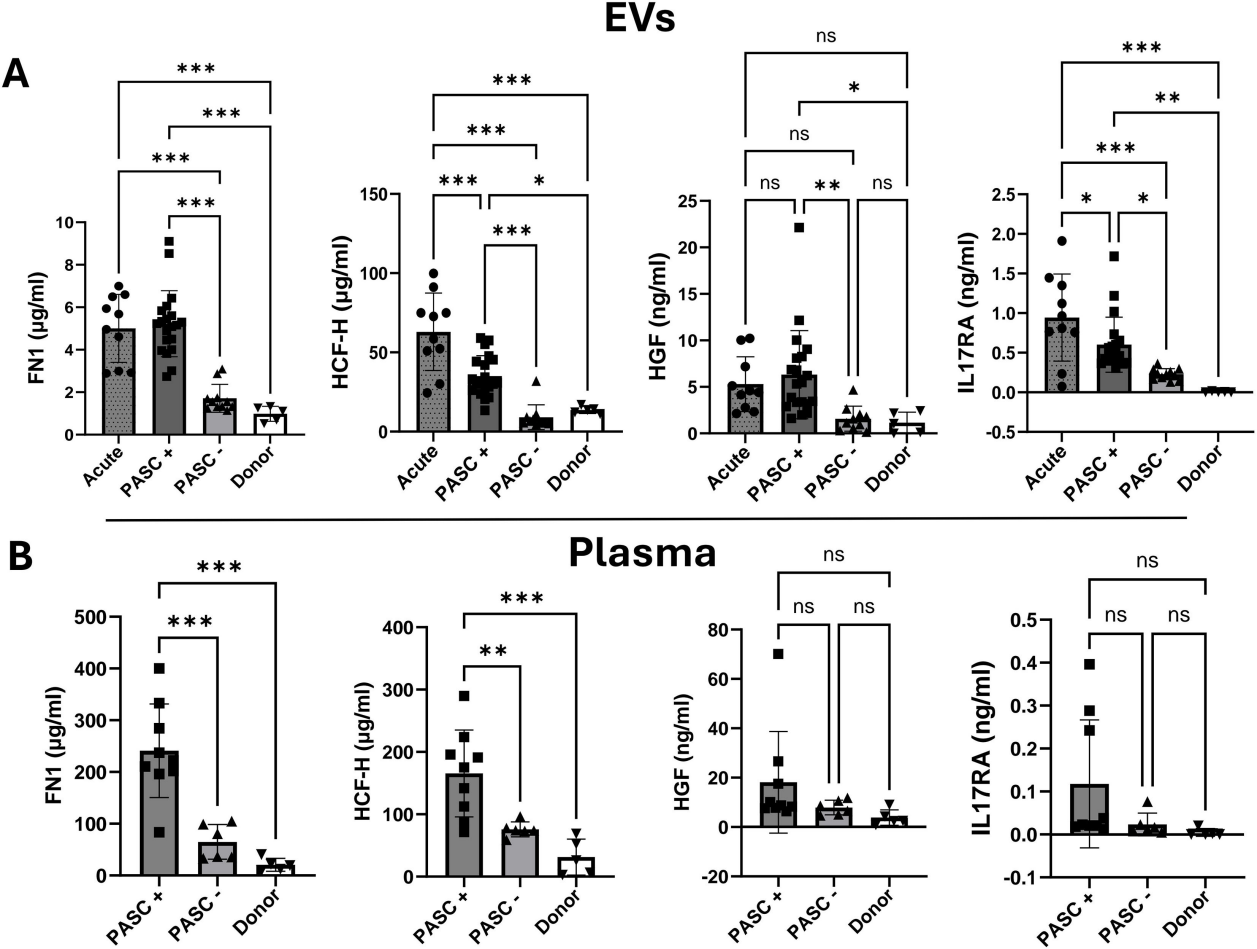
**(A)** Levels of FN1, HCF-H, HGF, and IL17RA in SEVs from Acute COVID-19 (n=10), PASC-positive ((n=20)), PASC-negative ((n=11)), and healthy individuals ((n=5)) using ELISA. **(B)** Comparison of FN1, HCF-H, HGF, and IL17RA levels across plasma from PASC-positive, PASC-negative, and healthy donor groups as measured by ELISA. p > 0.05; *p < 0.033; **p < 0.002; ***p < 0.001.

We next validated four of the significantly altered PASC-positive signature proteins, which were previously reported as dysregulated in SEVs from hospitalized individuals with acute COVID-19 (17), to confirm whether specific acute- phase protein signatures persist into the post-acute period and contribute to PASC. As shown in **Figure 6**, SEVs from PASC−positive individuals exhibited significantly elevated levels of FN1, HCF−H, HGF, and IL−17RA compared with both PASC−negative participants and healthy controls, as measured by ELISA. Consistent with prior observations, SEVs from acute COVID−19 patients also showed higher levels of these proteins relative to PASC−negative and healthy groups. Although HCF-H and IL-17RA levels declined significantly from the acute to the PASC positive phase, FN1 and HGF levels not only remained elevated but showed a slight further increase.

Parallel analysis of corresponding plasma samples showed that FN1 and HCF-H levels were significantly elevated in PASC-positive plasma compared with PASC-ve and healthy donors, while HGF and IL17RA showed no significant differences across groups (**Figure 6**). Although FN1, HGF, and HCF-H were present at substantially higher absolute concentrations in plasma, approximately 4-40-fold greater than in plasma-derived SEVs, SEV-based comparisons demonstrated better discrimination between PASC-positive and PASC-negative individuals. In particular, HGF and IL17RA exhibited significant alterations exclusively in SEVs but not in matched plasma samples, with higher concentrations of IL-17RA in SEVs than plasma, underscoring the enhanced sensitivity of SEV-associated proteins for distinguishing PASC status. Furthermore, the median fold difference for HCF-H between PASC-positive and PASC-negative groups was nearly twice in SEVs (4.6-fold) than in plasma (2.3-fold). Collectively, these findings suggest that SEVs may offer better discrimination of certain disease-associated molecular alterations compared with direct plasma analysis.

## Discussion

In this study, we compared the plasma-derived SEV-associated protein cargo of individuals with and without PASC and identified clear molecular differences that highlight inflammation, coagulation abnormalities, complement activation, and cell-cycle dysregulation. Persistent elevations of certain proteins previously dysregulated in acute COVID-19 were far more pronounced in SEVs than in matched plasma, with some showing significant changes only in the SEV compartment. These SEV-linked alterations, strongly differentiating PASC-positive individuals from PASC-negative individuals, underscore the importance of SEVs as indicators of ongoing pathophysiological processes.

Extracellular vesicles have been widely recognized as critical mediators in COVID-19 by contributing to both the hyperinflammatory and pro-thrombotic state that characterizes severe acute infection (27). Prior work, including our own, demonstrated that the plasma EVs from acute COVID-19 patients are enriched in pro-inflammatory and pro-thrombotic proteins such as tissue factor, complement components, and endothelial markers (17, 28, 29) and can directly trigger apoptosis in endothelial cells (17). Other studies similarly showed that circulating EVs from critically ill COVID-19 patients drive lung inflammation and promote macrophage polarization toward a pro-inflammatory M1 phenotype (30). Collectively, these findings establish EVs as active participants in acute COVID-19 pathogenesis.

Emerging evidence now extends the role of EVs in the pathophysiology of Long-COVID or PASC by acting as persistent carriers of viral and inflammatory molecules. EVs containing viral components may facilitate the systemic spread of the virus, evade immune surveillance, and allow delivery of viral proteins to cells without relying solely on conventional receptor-mediated entry mechanisms (31, 32). Our previous work demonstrated the persistent presence of SARS-CoV-2 spike protein within circulating SEVs from PASC patients up to a year or longer post-infection (33), while individuals without PASC showed decline or clearance. This persistent presence of spike-containing SEVs may serve as an ongoing trigger for immune dysregulation in PASC (33). In the present study, we used a high-throughput proteomic approach to understand the alterations in the SEV cargo associated with the development of PASC.

The SEV proteome of PASC-positive individuals revealed enrichment in proteins linked to inflammation, coagulation, and complement activation. These findings align well with multiple recent reports implicating persistent immune activation in PASC (34–38). Multiple proteins associated with apoptosis, cell cycle dysregulation, and lung injury were also elevated, consistent with plasma biomarker signatures described in PASC samples reporting breathlessness (36). Complement components such as C4b, FGG, and CFP, previously reported to be dysregulated during acute COVID-19 and contributing to thrombo-inflammation, were similarly elevated in PASC SEVs, further supporting the concept of a sustained complement-coagulation imbalance in long COVID (39–41). SEVs from individuals with PASC showed significantly higher levels of FN1, HCF-H, HGF, and IL-17RA compared with both PASC-negative participants and healthy controls. These findings mirror patterns previously observed in hospitalized patients during acute COVID-19 (17). While HCF-H, and IL-17RA levels dropped from the acute phase to the post-acute period, FN1 and HGF remained elevated, suggesting that certain acute-phase protein signatures persist and may contribute to ongoing PASC biology. This is even more interesting as the acute COVID-19 cohort that was compared for validation studies consisted entirely of hospitalized patients, whereas most of the individuals in the PASC cohort had mild initial infections. Altogether, the persistence of molecules implicated in acute COVID-19 biology within SEVs suggests a potential mechanistic link between EV cargo and the chronic features observed in PASC.

Furthermore, cytokines primarily involved in immune-cell maintenance, immunoregulatory signaling, and homeostatic immune responses, such as IFIT1, CSF2 (GM-CSF), IL-3, and IL-13 (42–45) were reduced in the PASC-positive group. IFIT1, also known as ISG56, which acts as a critical host anti-viral protein, including SARS-CoV-2 (46) was also markedly low in the PASC-positive SEVs. The coordinated downregulation of these proteins suggests altered immune-homeostatic signaling and compromised antiviral response in PASC-positive individuals.

Although numerous plasma- and serum-based proteomic studies have identified inflammatory, coagulopathic, and vascular proliferative pathways associated with PASC (37, 38, 47, 48), EV-based profiling may offer greater sensitivity for detecting persistent abnormalities. A notable multiplex study comparing plasma and plasma-derived EVs collected from COVID-19 survivors and uninfected controls 15 months after acute SARS-CoV-2 infection found elevated pro-inflammatory cytokines and neurofilament proteins in EVs but not in the plasma of COVID-19 survivors. Similarly, we observed stronger differences between PASC-positive and PASC-negative SEVs compared to matched plasma, particularly HGF and IL-17RA, which were significantly altered in SEVs but not in plasma. Additionally, IL-17RA levels were found to be higher in SEVs compared to plasma, and this could be because IL-17RA is a membrane-bound receptor protein, and therefore effectively detected in SEVs than in plasma. This suggests the importance of EVs in the persistence of immune activation post-COVID infection (49) and highlights the unique ability of EVs to capture subtle biological disturbances.

Circulating EVs have also been shown to mediate endothelial dysfunction in PASC, reinforcing their pathogenic contribution (50). EV protein profiling importance is further highlighted from a study comparing mitochondrial proteins in the plasma-derived EVs across uninfected individuals, acute-COVID, PASC-positive, and PASC-negative groups (51). This study demonstrated that abnormal levels of mitochondrial proteins in EVs during acute infection may serve as a predictor of future PASC development. Additionally, persistent alterations of these mitochondrial proteins in EVs from PASC -positive individuals were associated with neuropsychiatric symptoms (51).

Our symptom−specific analysis of SEV−associated proteins identified molecular patterns that were associated with particular PASC symptoms. Brain fog corresponded to higher levels of NOL4L, STK24, PRTN3, and IL17RA, suggesting involvement of stress−responsive signaling, apoptosis, neuroinflammation, and blood–brain barrier injury (52–56). Sleep disturbance was associated with increased IFIT1, AUNIP, METTL16, PPP3CA, and RGS22, pointing toward antiviral activity, cell−cycle regulation, RNA methylation, and cerebrospinal fluid-related homeostasis (45, 57–60).

These symptom−linked signatures suggest that SEVs reflect activation of inflammatory and stress−related pathways in PASC-positive individuals experiencing brain fog and sleep disturbances. However, these findings should be interpreted cautiously due to the study’s sample size and the multifactorial nature of symptoms that overlap with many inflammatory, neurological, and metabolic conditions. Another key limitation is that PASC-positive participants were identified through self-reported symptoms, introducing the possibility of misclassification. In addition, many participants had comorbidities, making it difficult to determine whether their symptoms reflected true PASC or simply the worsening of underlying chronic conditions.

Overall, our findings support the hypothesis that circulating SEVs contribute to PASC pathophysiology and carry biomarker candidates reflecting immune, vascular, and neurological abnormalities. Future studies in larger, clinically well-characterized cohorts, including comparisons between PASC individuals with and without organ-specific symptom clusters, will be essential to validate these associations. In conclusion, this study highlights a potential role of SEVs and their molecular cargo in the inflammatory, thrombotic, coagulopathic, and endothelial abnormalities characteristic of PASC. Targeting SEV-associated pathways or leveraging SEV cargo as minimally invasive biomarkers may ultimately improve diagnostic accuracy and guide therapeutic strategies for Long-COVID.

## Data Availability

Original datasets are available in a publicly accessible repository: This data can be found here: https://doi.org/10.5281/zenodo.21283519.
